# R3D Align web server for global nucleotide to nucleotide alignments of RNA 3D structures

**DOI:** 10.1093/nar/gkt417

**Published:** 2013-05-28

**Authors:** Ryan R. Rahrig, Anton I. Petrov, Neocles B. Leontis, Craig L. Zirbel

**Affiliations:** ^1^Department of Mathematics and Statistics, Ohio Northern University, Ada, OH 45810, USA, ^2^Department of Chemistry, Bowling Green State University, Bowling Green, OH 43403, USA and ^3^Department of Mathematics and Statistics, Bowling Green State University, Bowling Green, OH 43403, USA

## Abstract

The R3D Align web server provides online access to ‘RNA 3D Align’ (R3D Align), a method for producing accurate nucleotide-level structural alignments of RNA 3D structures. The web server provides a streamlined and intuitive interface, input data validation and output that is more extensive and easier to read and interpret than related servers. The R3D Align web server offers a unique Gallery of Featured Alignments, providing immediate access to pre-computed alignments of large RNA 3D structures, including all ribosomal RNAs, as well as guidance on effective use of the server and interpretation of the output. By accessing the non-redundant lists of RNA 3D structures provided by the Bowling Green State University RNA group, R3D Align connects users to structure files in the same equivalence class and the best-modeled representative structure from each group. The R3D Align web server is freely accessible at http://rna.bgsu.edu/r3dalign/.

## INTRODUCTION

Sequence alignment of biological macromolecules provides important information regarding their evolutionary histories, functional similarities and differences, and mechanisms of adaptation to new environments (1–4). Methods for sequence alignment of proteins and nucleic acids were first developed over 40 years ago and have been subject to improvement ever since (5–7). Methods for 3D structure alignment of RNA molecules are newer (8–14). The rapid increase in the number of atomic-resolution RNA structures in distinct functional states and from diverse sources provides new opportunities for understanding RNA function, evolution and adaptation at a finer grained level. To fully exploit these opportunities, robust methods are needed for generating 3D alignments that deal adequately with the challenges posed by the inherent flexibility of RNA, the conformational changes induced by crystal contacts and by ligand binding, the presence of disordered regions and unreliable modeling resulting from the use of low-resolution data.

### Previous results and comparison with other methods

Structural alignment of biological macromolecules is not a trivial task. Homologous molecules accumulate differences in sequence over evolutionary time. Although the 3D structures tend to change more slowly, significant structural differences can also accumulate over time. Because of computational challenges posed by the inherent difficulties noted earlier in the text, all RNA 3D alignment methods currently implemented on web servers, including ARTS, DIAL, SARA, iPARTS, SETTER and RNA 3D Align (R3D Align) (9–10,13,15–18), are based on heuristic approaches. Limitations to these approaches include alignment inaccuracies resulting from techniques to decrease runtime or restrictions on the sizes of the structures to be aligned. Although the SETTER web server is capable of quickly aligning the largest RNA structures, it does not produce alignments at the nucleotide level (18). R3D Align produces accurate nucleotide-to-nucleotide alignments in a reasonable amount of time, even for the largest ribosomal RNA structures.

In this article, we describe the features and use of the redesigned and enhanced R3D Align web server. The revamped R3D Align user interface includes weekly synchronization with the PDB database, more intuitive guidance for advanced parameter specification and integration with RNA 3D Hub for in-depth information about RNA 3D structures, chains and similar structures. The results page now features additional alignment summaries, including summary statistics and visual displays available for immediate viewing.

## MATERIALS AND METHODS

The R3D Align web server is built on the alignment algorithm described in (13). A brief overview is provided here. R3D Align decomposes each 3D structure into sets of local nucleotide neighborhoods, which typically overlap in the sense that each nucleotide is included in more than one set. The minimum number of neighborhoods that include each nucleotide is set by the parameter *p*. Pairs of neighborhoods, one from each structure, are superposed, and their geometric similarity is quantified using the geometric discrepancy, a measure first described in (19). This measure includes an RMSD term for nucleotide centers, as well as an angular term to quantify base co-planarity. A specially defined graph, known as the ‘local alignment graph’, is created that has vertices representing pairs of geometrically similar neighborhoods. Vertices in the local alignment graph are established for each pair of neighborhoods for which the geometric discrepancy is less than a user-specified threshold (parameter *d*) and whose sequence positions are closer than half the alignment bandwidth (parameter *β*). Edges are drawn between vertices in the local alignment graph, only when the local alignments they represent are compatible with each other. The global alignment is then constructed on the basis of a maximal clique of the local alignment graph. Further details regarding the setting of parameters *p*, *d*, and *β* are provided in next section.

## DESCRIPTION OF INPUT

**Figure 1.**
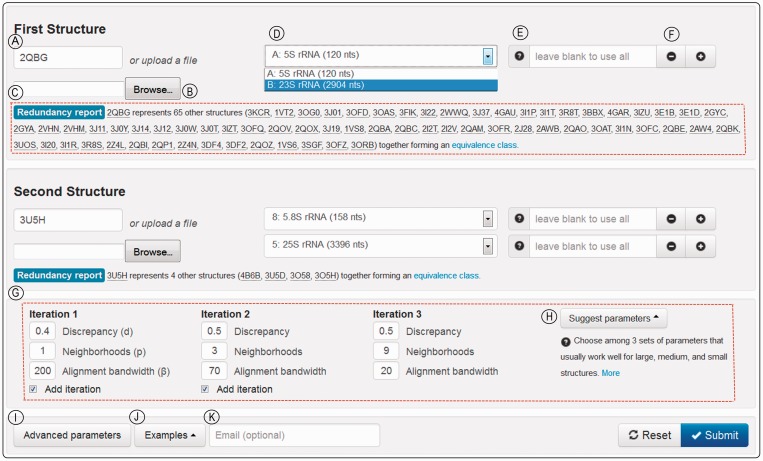
R3D Align web page for user input. Encircled capital letters label features. (**A**) Text box to specify the PDB code of the first structure to align; optionally, (**B**) shows where the user can upload a PDB-formatted file. (**C**) On entry of a PDB code, users are provided the redundancy report for the chosen structure, identifying the PDB code of the file that represents the structure class. (**D**) When users enter a PDB code, they are provided a drop-down list from which to select among the chains in the file. (**E**) Text box that allows users to restrict attention to one or more ranges of nucleotide numbers within the chain. The question mark by (E) explains how to enter nucleotide ranges. (**F**) The plus and minus signs allow users to add or remove sequence fragments. (**G**) The advanced parameter input box, hidden by default, is made visible by clicking the ‘Advanced Parameters’ button (**I**) and is initially populated with default parameters that work reasonably well for structures of all sizes. Optionally, the ‘Suggest parameters’ button (**H**) suggests parameters more efficient based on the size of the input structures. (**J**) The ‘Examples’ button provides complete input specifications for sample ribosomal alignments. (**K**) The user may provide an email address to be notified when the job is completed and receive the URL of the results page.

The input interface for R3D Align is shown in Figure 1 and is described in detail in the legend. Typical workflow occurs in this way: users specify two RNA 3D structures to align, select alignment parameters and submit the request to the server. Users can upload their own 3D structure files in PDB format or specify existing 3D structure files from the Protein Data Bank using the 4-character PDB ID. When the PDB ID is specified, R3D Align provides additional features: all RNA chains present in the PDB file are dynamically loaded into the input page to allow the user to specify chains and nucleotide ranges to align. To assist the user in typing PDB IDs, auto-completion has been implemented. The local cache of RNA 3D structures is synchronized weekly with the PDB database (20).

R3D Align uses the specified chain order and, as with sequence alignments, respects nucleotide order when producing an alignment; therefore, the order in which chains are requested for each file is important. The user may restrict attention to one or more ranges of nucleotide numbers within each chain, as desired. R3D Align is unique among RNA alignment servers in offering users the capability to align domains, motifs or other sub-structures comprising one or more non-contiguous segments of nucleotide sequence from one or more chains. Among other alignment servers, only DIAL allows for the specification of a subsequence, but this is limited to a single contiguous chain segment (9).

### Accessing example input

As a learning aid, the web server provides one-click loading of example input parameters (Figure 1J). On selecting the example and clicking the ‘Submit’ button, the user is immediately taken to a pre-computed Results page, discussed in detail later in the text.

### Input validation

To ensure that user-submitted input can be processed, extensive pre-submission input validation has been implemented, and users are informed of specific errors in their input. This includes nucleotide validation to avoid the server attempting queries specifying non-existent nucleotides.

### Use of representative structures

R3D Align has been integrated with RNA 3D Hub (in preparation), which provides users access to the equivalence classes of RNA PDB files described previously in (21). Each equivalence class comprises PDB files that contain 3D structures of the same type of RNA molecule from the same organism. Thus, all structures of *Escherichia coli* 16S rRNA belong to one equivalence class, all structures of *E. coli* 23S rRNAs to another and all *Thermus thermophilus* 16S rRNA structures to a third. When a user enters an existing PDB ID (Figure 1A), a list of all 3D structures belonging to the same equivalence class appears (Figure 1C), and the user is informed which PDB file contains the best-modeled representative of that class, according to the criteria implemented to define the non-redundant sets of RNA structures used by RNA 3D Hub (21).

### Alignment parameters

Users can choose to keep the default alignment parameters or modify them by clicking the ‘Advanced Parameters’ button (Figure 1I) and then entering alternative values in the input boxes indicated Figure 1G. Advanced alignment parameters under user control include *d*, *p*, *β* and the number of iterations to be carried out. The default values for these parameters have been optimized through a series of rigorous tests of thousands of alignments. The default parameters provided are suitable for structures of all sizes. Parameter values customized to the size of the structures selected by users can be accessed by clicking the ‘Suggest parameters’ button (Figure 1H). Applying default or customized parameters typically decreases the alignment runtime without compromising alignment quality, compared with other potential parameter choices.

### Iterative alignment

The R3D Align web server has been designed to iteratively produce structural alignments to achieve more efficient processing and higher accuracy. In the iterative approach, the alignment produced in one iteration is used as a seed alignment for the subsequent iteration. With an iterative approach, structures larger than 150 nt can be aligned in a significantly shorter period, without compromising the quality of the alignment. R3D Align uses an iterative approach by default. The initial seed alignment is determined by a simple dynamic programming sequence alignment, which may be inadequate for significantly diverged homologues. In this case, increasing the bandwidth parameter (*β*) is advised.

In general, in carrying out successive iterations, the user should progressively increase the value of *p* while decreasing the value of *β* as described in the Help section of the website. Typical values of *p* range from 1 to 10. Increasing *p* increases runtime, but typically also increases alignment accuracy. Regarding the geometric discrepancy cutoff parameter, *d*, typical values lie between 0.3 and 0.6. Higher values of *d* allow regions that are less geometrically similar to be aligned but require longer runtime, as this includes more pairs of neighborhoods in the local alignment graph. More specific details and guidance regarding setting parameter values can be found in the extensive Help section that accompanies the web server.

## DESCRIPTION OF OUTPUT

Immediately on job submission, R3D Align generates a unique web address to display the results. This page displays the job status until it is completed. The R3D Align Results page provides interactive access to textual and visual displays of the computed alignment. All results can also be downloaded for local use. When an email address is entered on the input page, the user is sent notification of job completion and the link to the Results page.

**Figure 2.**
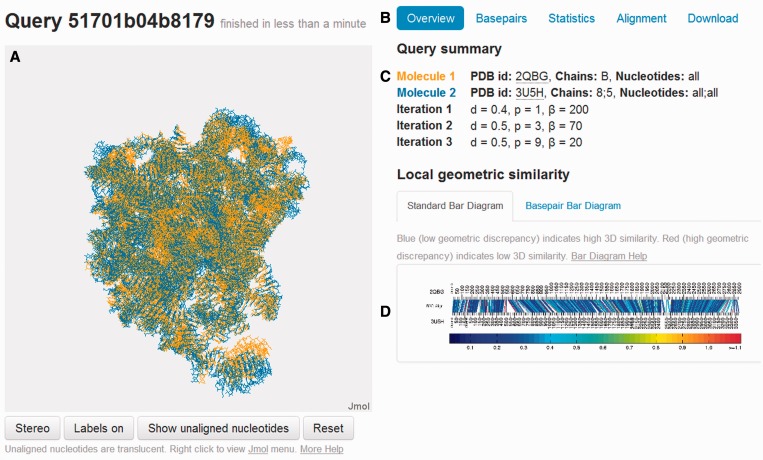
R3D Align Results page. Labels in encircled capital letters indicate the following features. (**A**) Jmol applet displaying the rigid-body superposition of the two aligned structures. (**B**) Tabs from which a variety of displays of the alignment can be accessed. (**C**) Input parameters for the alignment. (**D**) Bar Diagram accessible with the Overview tab.

Notably, when users submit previously processed queries, the alignments are retrieved from a database of completed jobs, and users are immediately taken to results pages without delay. All results are stored on the server indefinitely with stable URLs, to facilitate collaboration, sharing and archiving results. This feature is used to generate the Gallery of Featured Alignments, described later in the text. URLs include a long randomly generated text string, revealed only to the user, to protect the privacy and security of user data and analyses. Figure 2 provides an example of the R3D Align Results page.

On the left side of the Results page (Figure 2A), the alignment is visualized approximately as a rigid-body superposition of the 3D structures using an interactive Jmol applet (http://jmol.org). The applet displays aligned nucleotides in opaque colors, orange for structure 1 and blue for structure 2, which conform to the guidelines suggested in (22).

Nucleotides that are not aligned by R3D Align are displayed in translucent orange and blue and can be toggled on and off in the display. The color scheme is indicated in the input summary (Figure 2C). Additional visualization options can be accessed by right-clicking in the Jmol window. Readers should note that the rigid-body superposition necessarily only approximates the locally optimized flexible alignments generated by R3D Align.

The right side of the Results page features summary output of the alignment in a variety of formats. The user can toggle between the different displays using the tabs at the top (Figure 2B). All output is directly accessible on the Results page for viewing and interaction; files can also be downloaded for local use.

The default Overview tab (Figure 2B) provides a summary of the query (Figure 2C) along with a unique and informative display of the alignment that we call the ‘Bar Diagram’. The standard Bar Diagram (Figure 2D) provides a concise yet detailed visual summary of the alignment at the individual nucleotide level color coded by local structural similarity. The nucleotide numbers of the first structure are arrayed across the upper horizontal line of the Bar Diagram and the nucleotides of the second structure along the lower horizontal line. Aligned nucleotides are joined by line segments. Each line segment is colored to indicate how well the local neighborhoods superimpose in 3D space. Specifically, for each nucleotide in the first structure that has a corresponding nucleotide in the second, the four nearest nucleotides with a correspondence in the other structure are found, and the geometric discrepancy between the corresponding five nucleotides in the two structures is computed to color the line. As indicated by the color bar below each Bar Diagram, discrepancy values are color coded from blue, for low discrepancy (high structural similarity), to red, for high discrepancy (low structural similarity).

The Basepair Bar Diagram tab presents an enhanced version of the Bar Diagram that displays the basepairing interactions, as annotated by FR3D (23), in each structure as arcs colored by basepair type and topology. Basepair diagrams are similar to the ‘double structure arc diagrams’ used in (24) to evaluate secondary structure predictions and alignments and those used in (25) to visualize RNA base-pairing probabilities. Figure 4 displays three Basepair Bar Diagrams. The R3D Align Help pages contain numerous examples to aid in the interpretation of Bar Diagrams.

Although structural alignment of proteins focuses on optimizing the alignment at the residue level, alignment of RNA structures requires, in addition, that basepairs be correctly aligned. To assess the quality of the RNA structural alignments produced by R3D Align, basepair alignments are provided in tabular form under the Basepairs tab (Figure 2B). Unlike conventional horizontal sequence alignments, basepair alignments are provided vertically in the Basepair Table, with each row corresponding to an aligned basepair in the structure. Nucleotides that form more than one basepair appear in multiple rows. Nucleotides forming no basepairs appear only once.

**Figure 3.**
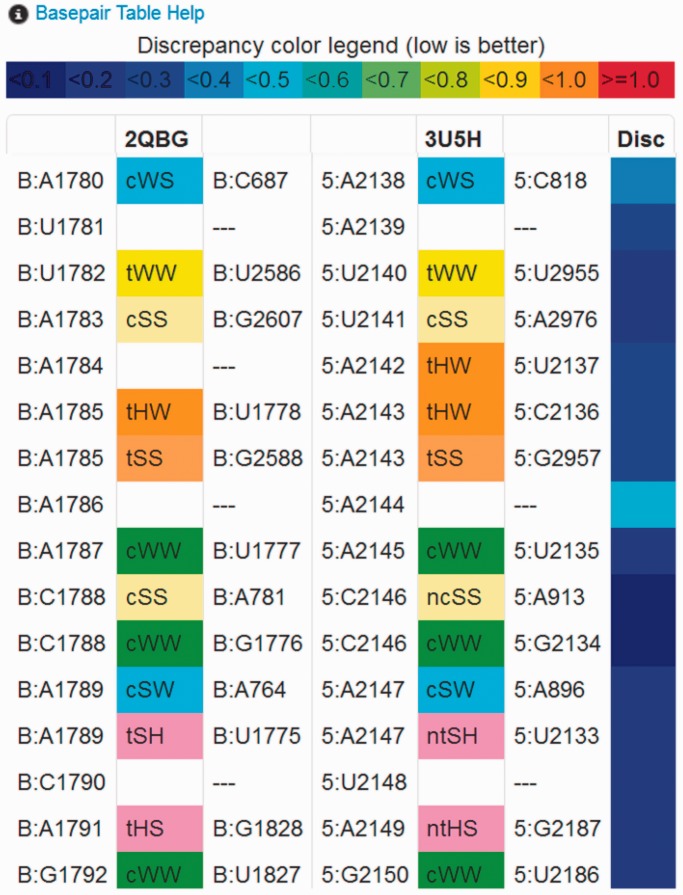
Sample Output: Basepair Table. The Basepair Table, accessible under the Basepairs tab (Figure 2B), lists all the nucleotides and annotated basepairs of the two aligned structures sequentially. Columns 1–3 list nucleotides and pairs of the first aligned structure (chain:base_type nt_number) and columns 4–6, those of the second. Nucleotides and basepairs that are aligned by R3D Align appear in the same row. Basepair types (26) are listed in columns 2 and 5 and are color coded to facilitate visual comparison of local basepair correspondences. When one structure has a basepair aligned with two non-basepairing nucleotides in the other structure, another pairwise interaction type may be listed. ‘Near’ basepair annotations are prefixed with ‘n’ and colored in the same way as the corresponding pairs. Stacking interactions are prefixed by ‘s’. Column 7 indicates the local geometric discrepancy for the nucleotides in Columns 1 and 4, as calculated for the Bar Diagrams.

Figure 3 provides a small portion of the Basepair Table for the alignment displayed in Figure 2, corresponding to a region of high structural similarity, where nearly all basepairs in one structure are aligned to basepairs of the same geometric type in the second structure. Moreover, the local geometric discrepancies calculated for corresponding nucleotides in the two structures are low (blue colors in Column 7). Although, nucleotides U1781, A1784, A1786 and C1790 in 2QBG form no basepairs, each is aligned to a nucleotide in 3U5H.

The Statistics tab presents numerical summaries of the structures and alignment. The Alignment tab (Figure 2B) displays a conventional (horizontal), interleaved sequence alignment of the nucleotides from the two structures. The user can download the alignment in FASTA format by clicking the Download tab. The Download tab also provides access to the Bar Diagrams in pdf format and the Basepair Table in csv format. Columns 1 and 4 of the Basepair Table can be used to produce a list of all nucleotide-to-nucleotide correspondences between the two structures, which may be more helpful than the alignment in FASTA format for certain purposes.

## GALLERY OF FEATURED ALIGNMENTS

To facilitate use of the R3D Align server, we have pre-computed pairwise alignments of ribosomal RNA structures using the representative structures of their equivalence classes (21). The rRNA molecules that are featured in the Gallery and their sources are listed in Table 1.

**Table 1.** Molecules and organisms that appear in the Gallery of Featured Alignments

Molecules	Organisms
SSU rRNA (16S and 18S ribosomal RNA)	Bacteria: E. coli, T. thermophilus
	Eukarya: Saccharomyces cerevisiae, Thermomyces lanuginosis
LSU rRNA (23S and 26S ribosomal RNA)	Bacteria: E. coli, Deinococcus radiodurans, T. thermophilus;
5S rRNA	Archaea: H.marismortui; Eukarya: S. cerevisiae, T. thermophila

The Gallery can be accessed from the top of the R3D Align input page. For each class of rRNA molecule (5S, SSU and LSU rRNA), the representative structures from each organism are aligned pairwise. The Gallery page features a table with images of the rigid-body superpositions for each pair of aligned molecules. Clicking an image takes users to the pre-computed R3D Align Results page for that pair of structures. The content of these pages is described under Description of Output.

The nucleotide-to-nucleotide alignments of 3D structures produced by R3D Align display features not evident in conventional multiple sequence alignments, including the degree of conservation of local geometry, as evidenced by the types of non-Watson–Crick basepairs. Previous work documented that the types of non-WC basepairs are highly conserved in the rRNAs of evolutionarily distant bacteria (*E. coli* and *T. thermophilus*) and archaea (*Haloarcula marismortui*) (27). The Gallery of Featured Alignments allows one to explore the structural similarities and differences at the basepair and motif level between the bacterial, archaeal and eukaryal rRNAs.

## EXAMPLE USE CASE FOR R3D ALIGN

An additional use case for R3D Align is the evaluation of results from RNA 3D structure prediction software by alignment of modeled structures to experimental structures. Comparison of predicted models with known structures using rigid-body superposition and calculation of RMSD can be misleading, owing to the inherent flexibility of RNA. Using R3D Align, one obtains locally optimized alignments, which can be visualized with the unique diagnostic tools provided by the R3D Align web server, which facilitates assessing 3D alignment quality. To provide a real-world example, we focus on Challenge 3 from the recent RNAPuzzles structure prediction competition—prediction of the 3D structure of a riboswitch domain solely from sequence (28).

**Figure 4.**
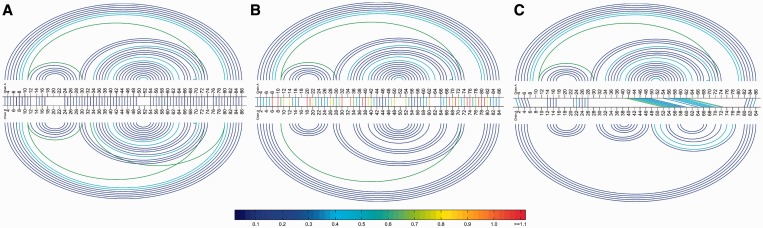
Comparison of alignments of good, medium and low quality models to the experimentally determined structure, using the R3D Align Basepair Bar Diagram. Aligned nucleotides are indicated by straight colored lines linking the two nucleotide sequences, color coded by geometric discrepancy. The arcs above and below correspond to basepairs in the first and the second structures, respectively. Nested cWW basepairs belonging to the 2D structure are colored royal blue. Non-nested cWW basepairs, forming pseudoknots, when present, are colored red. Nested non-cWW basepairs, belonging to hairpin and internal loops, are colored light blue, and non-nested non-cWW pairs, forming tertiary contacts, are colored green. (**A**) 3D alignment of two versions of the same experimentally determined 3D structure representing the solution for Challenge 3 of the RNAPuzzles competition (28). (**B**) 3D alignment of lowest RMSD structure (below) with an experimentally determined structure (above). (**C**) 3D alignment of highest RMSD structure (below) with an experimentally determined structure (above). Both predicted structures lack most of the non-Watson–Crick basepairs observed experimentally. These diagrams can be accessed online using the URLs listed in Supplementary Table S1.

Figure 4 shows three Basepair Bar Diagrams comparing predicted and experimental structures. Panel A shows the alignment of two structures calculated from the experimental X-ray crystallography data so as to represent the range of models consistent with the experimental data. As expected, the geometric discrepancies in the bar diagram are low and the basepairs in the two structures match up well.

Panel B shows the *lowest* RMSD (7.24 Å) model from the RNA Puzzles competition, the one submitted by the Chen group. The arcs indicate that the overall architecture is accurately captured and most Watson–Crick basepairs are identified, but some non-Watson–Crick basepairs are lacking. The colors of the line segments indicate there are several regions with high geometric discrepancy.

For purposes of illustration, Panel C shows the *highest* RMSD (22.99 Å) model, submitted by the Major group. Few nucleotides can be aligned properly, and the secondary structure is different in one part of the model structure.

Supplementary Table S1 lists URLs to the alignments in Figure 4 and to the original RNAPuzzles results pages. R3D Align output pages provide a permanent means for compiling results of prediction competitions like RNAPuzzles and related purposes because all R3D Align results are stored indefinitely with stable URLs.

## IMPLEMENTATION

R3D Align computations are performed by a suite of programs written in Matlab. The web application uses PHP, Javascript, Perl and MySQL and is implemented using open-source industry-standard frameworks, including CodeIgniter (http://codeigniter.com), jQuery (http://jquery.com) and Twitter Bootstrap (http://twitter.github.com/bootstrap). The source code for both the standalone R3D Align program and the web application can be found at https://github.com/BGSU-RNA/. The server runs on a Mac Pro with a 2.66 GHz Quad-Core Intel Xeon processor and 6 Gb of RAM. Up to four jobs can be processed simultaneously, but if an alignment takes longer than 30 min of CPU time, the job is terminated. R3D Align processed >1800 queries between November 2010 and April 2013. According to Google Analytics, the site had 377 unique visitors from 46 countries over the same period. The web interface has been tested on Chrome, Safari and Firefox browsers on Mac OS X and Firefox and Chrome on PC. R3D Align is hosted and maintained by the Bowling Green State University RNA Bioinformatics group. Questions or feedback can be directed using the form on the website or by submitting a bug report via GitHub.

## SUPPLEMENTARY DATA

Supplementary Data are available at NAR Online: Supplementary Table S1.

## FUNDING

Funding for open access charge: National Institutes of Health [5R01GM085328-03 to N.B.L. and C.L.Z.].

*Conflict of interest statement.* None declared.
